# Lipopeptide ligands captured by MHC class I molecules undergo dynamic conformational changes that affect their antigenic strength

**DOI:** 10.1016/j.jbc.2025.111049

**Published:** 2025-12-12

**Authors:** Daisuke Morita, Toshiki Fujii, Shinsuke Inuki, Hiromu Suzuki, Bunzo Mikami, Masahiko Sugita

**Affiliations:** 1 Laboratory of Cell Regulation, Institute for Life and Medical Sciences, Kyoto University, Kyoto, Japan; 2 Laboratory of Bioorganic Medicinal Chemistry, Graduate School of Pharmaceutical Sciences, Kyoto University, Kyoto, Japan; 3 Laboratory of Bioorganic Medicinal Chemistry, Graduate School of Biomedical Sciences, Tokushima University, Tokushima, Japan; 4 Laboratory of Medicinal Chemistry, Institute of Photonics and Human Health Frontier, Tokushima University, Tokushima, Japan; 5 Laboratory of Cell Regulation and Molecular Network, Graduate School of Biostudies, Kyoto University, Kyoto, Japan; 6 Laboratory of Metabolic Science of Forest Plants & Microorganisms, Research Institute for Sustainable Humanosphere, Kyoto University, Kyoto, Japan; 7 Structural Energy Bioscience, Institute of Advanced Energy, Kyoto University, Kyoto, Japan

**Keywords:** antigen presentation, immunology, lipopeptide antigen, major histocompatibility complex class I, protein myristoylation, T-cell receptor, X-ray crystallography

## Abstract

A fraction of the major histocompatibility complex class I proteins can bind *N*-myristoylated short lipopeptides rather than conventional long peptides. The molecular mechanisms underlying lipopeptide antigen presentation were recently delineated for *N-*myristoylated 4-mer lipopeptides (C14-Gly1-Gly2-Ala3-Ile4; C14nef4) derived from the retroviral Nef protein. The C14nef4 lipopeptides are captured by the rhesus major histocompatibility complex class I allomorph, Mamu-B∗05104, and recognized by specific αβ T-cell receptors (TCRs). The crystal structure of the Mamu-B∗05104–C14nef4–TCR complex indicates that both ends of C14nef4, namely, myristic acid and C-terminal Ile4, are anchored at the antigen-binding groove, leaving Gly1, Gly2, and Ala3 exposed. Among these residues, only the amide bond of Gly1 forms a hydrogen bond with TCRs and serves as a primary T-cell epitope. However, it remains unclear how antigenic and nonantigenic lipopeptides exist, both of which share the primary T-cell epitope. To gain insight into this enigma, we utilized C14nef4 and its analogs with an amino acid substitution for Ala3. Biolayer interferometry experiments with immobilized TCRs and lipopeptide-bound Mamu-B∗05104 indicated that the antigenic strength varied among these lipopeptides. The crystal structures of Mamu-B∗05104 complexed with either C14nef4 or each of its five analogs showed a downward shift in the proximal part (C_1_–C_4_ carbons) of the hydrocarbon chain and the linked Gly1 residue for poorly antigenic analogs. Furthermore, molecular dynamics simulations indicated that lipopeptide ligands alter their conformation dynamically, with differential efficiency in exposing Gly1 externally. Thus, the antigenic strength of lipopeptides is affected by their intrinsic ability to sustain a T-cell epitope–exposed configuration.

Major histocompatibility complex (MHC) class I molecules bind peptide antigens and present them to CD8^+^ cytotoxic T lymphocytes that play a pivotal role in the host defense against viral infections and cancer (1, 2). The crystal structures of peptide-bound MHC class I complexes and those conjugated with specific αβ T-cell receptors (TCRs) have been extensively studied over the past 3 decades. These studies have elucidated how peptide antigens are captured by MHC class I molecules and recognized by TCRs at a molecular level (3, 4). The membrane-distal α1 and α2 domains of MHC class I heavy chains form a β-sheet platform topped by two semiparallel α-helices to create a groove for binding antigens, in which 8- to 11-mer peptide ligands are accommodated. The MHC class I antigen-binding groove contains six pockets, designated A-to F-pockets. The B- and F-pockets are used as primary anchoring sites for the second and C-terminal amino acid residues of peptide ligands, respectively. This leaves some of the central amino acid residues exposed for potential interactions with TCRs. TCR αβ heterodimers dock onto the MHC class I–peptide complex and survey the fine structure primarily using three complementarity-determining region (CDR) loops of each TCRα and β chain. Among these, the highly variable CDR3 loops that are generated as a result of the clonotypic V(D)J recombination of TCR genes play an essential role in the specific recognition of peptide ligands. The germline-encoded CDR1 and CDR2 loops also interact with polymorphic amino acid residues of the α1/α2 helices and contribute to MHC class I restriction (5).

The established paradigm of MHC class I–mediated peptide antigen presentation may now require some modifications to incorporate the novel MHC class I function of lipopeptide antigen presentation (6). The ability of MHC class I– and MHC class I–like proteins to bind *N*-myristoylated short lipopeptides rather than conventional long peptides has been observed in rhesus monkeys (Mamu-B∗05104 and Mamu-B∗098) (7, 8, 9), humans (HLA-A∗24:02 and HLA-C∗14:02) (10) and chickens (YF1∗7.1) (11). Importantly, the Mamu-B∗05104-restricted CD8^+^ T-cell line SN45 was established from the peripheral blood of a rhesus monkey infected with simian immunodeficiency virus. The SN45 T cells specifically recognize the N-terminal 4-mer lipopeptide fragment (C14-Gly1-Gly2-Ala3-Ile4; C14nef4) derived from the N-myristoylated simian immunodeficiency virus Nef protein (12). In addition, the ability of Mamu-B∗098 to present *N*-myristoylated 5-mer lipopeptides (C14-Gly1-Gly2-Ala3-Ile4-Ser5; C14nef5) to CD8^+^ cytotoxic T lymphocytes was demonstrated using Mamu-B∗098 transgenic mice deficient in TAP peptide transporter function (13). These findings suggest that a subset of MHC class I molecules can induce lipopeptide-specific T-cell responses that represent a unique immune pathway distinct from conventional peptide-specific T-cell responses.

We recently determined the crystal structure of the C14nef4-bound Mamu-B∗05104 complex in a form that is coupled with the SN45 TCR (14). The overall structure of Mamu-B∗05104 was comparable to that of conventional peptide-presenting MHC class I molecules, and the C14nef4 lipopeptide ligand is accommodated in the antigen-binding groove. The distal two-thirds of myristic acid and the C-terminal Ile4 residue of C14nef4 anchor at the B- and F-pockets, respectively, leaving the A-pocket unoccupied. Accordingly, the Gly1, Gly2, and Ala3 residues of the peptide portion, as well as the proximal part of the fatty acid, are exposed externally. However, only the amide bond of Gly1 interacts with the SN45 TCR by establishing a hydrogen bond with the glutamic acid residue at position 101 of the CDR3β loop. This structural analysis suggests that the unique amide bond of the *N*-myristoylated Gly1 residue present in *N*-myristoylated lipopeptide ligands but not in conventional peptides serves as the primary T-cell epitope used for lipopeptide recognition. Given that the amide bond of Gly1 is shared among all *N*-myristoylated lipopeptides, the mechanisms by which antigenic and nonantigenic lipopeptide ligands can be distinguished remain unclear.

J.RT3, a TCR-deficient Jurkat cell line, was transfected with SN45-derived TCRα and TCRβ complementary DNAs in a previous study. The SN45 TCR-reconstituted T-cell line (J.RT3/SN45) was used as responder T cells to compare antigenic activities among C14nef4-related analogs. In this cell-based analysis, C14nef4 and its analogs with an amino acid substitution at position 3 were unexpectedly separated into highly antigenic (C14nef4, C14-GGSI, and C14-GGKI) and poorly antigenic (C14-GGGI, C14-GGEI, and C14-GGII) groups (14). Among the lipopeptides of the highly antigenic group, a particularly enhanced antigenic activity was observed for the C14-GGSI analog with an EC_50_ approximately 10-fold lower than that of the authentic C14nef4 antigen. Therefore, we hypothesized that this simple set of lipopeptide ligands with differential antigenic activities might be valuable for elucidating the mechanisms underlying the regulation of lipopeptide antigenicity. In the present study, we used a series of biophysical (biolayer interferometry), structural (crystallography), and computational (molecular dynamics simulations) approaches for these lipopeptides. We found that lipopeptides accommodated in the antigen-binding groove of Mamu-B∗05104 were prone to dynamically alter their conformation, and the efficiencies in adopting the T-cell epitope–exposed conformation differed significantly between lipopeptide ligands.

## Results

### Intermolecular interactions between the SN45 TCR and lipopeptide-bound Mamu-B∗05104 complexes

**Figure 1:**
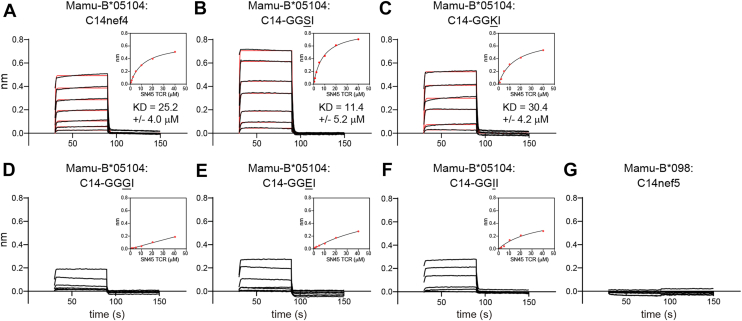
**Biolayer interferometry analysis of interactions between the SN45 TCR and lipopeptide-bound Mamu-B∗05104 complexes.** The molecular interaction kinetics of SN45 TCR and lipopeptide-bound Mamu-B∗05104 complexes were assessed using the Octet RED96 system, in which biotinylated SN45 TCR proteins were immobilized on streptavidin-coated sensors, and serially diluted Mamu-B∗05104 complexes were applied as analytes. Sensorgrams and the global curve fit (*red*) show Mamu-B∗05104 complexed with highly (C14nef4 [*A*], C14-GGSI [*B*], and C14-GGKI [*C*]) and poorly (C14-GGGI [*D*], C14-GGEI [*E*], and C14-GGII [*F*]) antigenic lipopeptides. The C14nef5-bound Mamu-B∗098 complex was prepared and used as a negative control (*G*). The steady-state affinity plots (*inset*) are also shown. Data are representative of three independent experiments, and mean *K*_*D*_ values ± SD are shown. Note that *K*_*D*_ values were not determined for poorly antigenic lipopeptides (*D–F*) because of poor global fitting. TCR, T-cell receptor.

T-cell assays using J.RT3/SN45 as responder cells suggested that C14nef4 and its five analogs could be separated into highly and poorly antigenic groups. Cell-based analyses may be influenced by undefined factors related to the use of live cells and serum components. Therefore, we first sought to validate the separation of the two groups at the molecular level. To this end, we investigated the kinetics of TCR–lipopeptide interactions in biolayer interferometry experiments. The ectodomain of the Mamu-B∗05104 heavy chain and rhesus β2-microglobulin (β2m) were produced in *Escherichia coli* as inclusion bodies, and the purified recombinant proteins were refolded in the presence of either C14nef4 or each of the five analogs with an amino acid substitution for Ala3. All refolded complexes were purified by size-exclusion and anion-exchange chromatography, and nearly identical elution profiles were obtained for each lipopeptide sample. A biotin-tagged form of the dimerized SN45 TCRα and β ectodomains was also prepared and immobilized using streptavidin-coated sensors in biolayer interferometry experiments, in which serially diluted Mamu-B∗05104–lipopeptide complexes were used as analytes. The C14nef4-bound Mamu-B∗05104 complex interacted with the SN45 TCR with a reasonable affinity (*K*_*D*_ = 25.2 ± 4.0 μM) that was comparable with that for conventional peptide-bound MHC–TCR interactions (Fig. 1*A*) (15). Contrastingly, the negative control C14nef5-bound Mamu-B∗098 complex failed to bind the SN45 TCR (*G*). The results for the five C14nef4 analogs (*B*–*F*) were consistent with the conclusion drawn by the cell-based analysis with J.RT3/SN45. First, lipopeptide samples were separated into highly antigenic (C14nef4, C14-GGSI, and C14-GGKI; *upper panels*) and poorly antigenic (C14-GGGI, C14-GGEI, and C14-GGII; *lower panels*) groups. Second, the C14-GGSI-bound Mamu-B∗05104 complex exhibited a higher affinity for the SN45 TCR (*K*_*D*_ = 11.4 ± 5.2 μM) (*B*) than that observed for the C14nef4-bound Mamu-B∗05104 complex (*A*). Taken together, these molecular and cell-based analyses consistently indicated the differential antigenic strength of lipopeptide ligands.

### Crystal structure of the SN45 TCR–C14-GGSI-bound Mamu-B∗05104 complex

**Figure 2:**
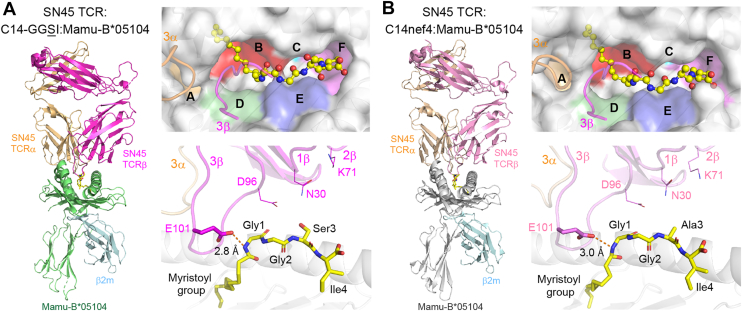
**SN45 TCR-docked structures of the C14nef4- and C14-GGSI-bound Mamu-B∗05104 complexes.** The overall structures of either C14-GGSI-bound (*A*) or C14nef4-bound (*B*) Mamu-B∗05104 heavy chain–β2m complexes and the associated SN45 TCRα and β heterodimer are shown (*left panels*). The top surface of the antigen-binding groove and alignment of the six pockets are shown in the *upper right panels* with bound ligands (*yellow sticks*). The CDR3α (*orange*) and CDR3β (*magenta*) loops of SN45 TCR are shown as *ribbon diagrams*. Side views of the TCR–ligand interface are shown in the *lower right panels* with bound lipopeptides and nearby CDR loops. *Dotted lines* indicate hydrogen bonds between the side chain of E101 of CDR3β and the nitrogen atom of the amide bond of the *N*-myristoylated Gly1 residues, and the hydrogen bond distance is provided. The structure of the C14nef4-bound Mamu-B∗05104–SN45 TCR complex was determined previously (Protein Data Bank ID: 7BYD) and used for comparison purposes. CDR, complementarity-determining region; TCR, T-cell receptor.

Given the enhanced antigenic strength observed for C14-GGSI compared with that of the authentic C14nef4 antigen, we initially hypothesized that besides the shared primary T-cell epitope (the amide bond of the Gly1 residue), the side chain of Ser3 of C14-GGSI may act in an additive manner by establishing direct interactions with the SN45 TCR. Therefore, the SN45 TCR–associated Mamu-B∗05104–C14-GGSI complex was crystallized, and the structure was determined at a resolution of 2.7 Å (Table 1). As shown in Figure 2*A*, the SN45 TCR αβ heterodimer docked onto the top surface of Mamu-B∗05104 (*left panel*), which accommodated the C14-GGSI lipopeptide in its antigen-binding groove. The B- and F-pockets interacted with the acyl chain and C-terminal Ile4 residue of C14-GGSI, respectively (*right upper panel*), and the epitopic amide bond of Gly1 was externally exposed (*right lower panel*). These features were virtually identical to those of the authentic C14nef4 antigen (Fig. 2*B*), which we previously reported (14). The spatial alignment of the TCR CDR loops was also nearly indistinguishable in both complexes, sustaining the critical hydrogen-bond interaction between the side chain of glutamic acid (E101) at position 101 of the CDR3β loop and the epitopic amide bond of Gly1 of the lipopeptide ligand (Fig. 2, *A* and *B*, *right lower panels*). Importantly, the CDR loops of the SN45 TCR were positioned far beyond the range of hydrogen bond formation with the side chain of Ser3 (Fig. S1). Therefore, it appears unlikely that an additional T-cell epitope besides the shared primary T-cell epitope was provided after the Ala3 to Ser3 substitution.

**Table 1:** Data collection and refinement statistics (molecular replacement)

	Mamu-B∗05104–C14nef4	Mamu-B∗05104–C14-GGSI	Mamu-B∗05104–C14-GGKI	Mamu-B∗05104–C14-GGGI	Mamu-B∗05104–C14-GGEI	Mamu-B∗05104–C14-GGII	SN45 TCR–Mamu-B∗05104–C14-GGSI
PDB ID	9WJ2	9WJ4	9WJ5	9WJB	9WJC	9WJD	9WNT
Data collection							
Space group	*P2*_*1*_*2*_*1*_*2*_*1*_	*P2*_*1*_*2*_*1*_*2*_*1*_	*P2*_*1*_*2*_*1*_*2*_*1*_	*P2*_*1*_*2*_*1*_*2*_*1*_	*P2*_*1*_*2*_*1*_*2*_*1*_	*P2*_*1*_*2*_*1*_*2*_*1*_	*P2*_*1*_
Cell dimensions							
a, b, c (Å)	53.07, 81.95, 104.22	52.95, 82.10, 106.54	52.97, 82.81, 107.70	53.13, 81.21, 107.40	53.04, 81.81, 107.49	48.84, 76.96, 127.03	84.22, 94.78, 123.19
β (°)	90	90	90	90	90	90	90.5
Resolution (Å)	50–2.15 (2.28–2.15)^a^	50–2.04 (2.16–2.04)	50–1.50 (1.59–1.50)	50–2.10 (2.23–2.10)	50–2.00 (2.12–2.00)	50–1.70 (1.81–1.70)	50–2.70 (2.86–2.70)
*R*_merge_	0.100 (0.672)	0.075 (0.546)	0.056 (0.487)	0.139 (0.444)	0.115 (0.509)	0.039 (0.375)	0.095 (0.865)
I/σ(I)	13.9 (3.1)	17.2 (3.88)	16.7 (2.46)	9.85 (3.67)	12.8 (4.10)	25.7 (3.14)	13.9 (1.48)
Completeness (%)	99.9 (99.4)	99.8 (99.1)	97.1 (84.6)	99.9 (99.8)	99.7 (98.7)	97.5 (86.9)	99.0 (94.9)
Redundancy	8.9 (8.8)	8.9 (8.9)	5.8 (4.5)	6.6 (6.8)	7.5 (7.8)	5.3 (4.2)	5.5 (4.3)
CC(1/2) (%)	99.8 (89.4)	99.8 (92.1)	99.8 (86.0)	99.3 (92.6)	99.7 (93.1)	100 (88.4)	99.7 (57.8)
Refinement							
Resolution (Å)	2.15 (2.23–2.15)	2.04 (2.10–2.04)	1.50 (1.52–1.50)	2.10 (2.17–2.10)	2.00 (2.06–2.00)	1.70 (1.73–1.70)	2.70 (2.75–2.70)
No. of reflections	25,397 (2761)	30,254 (2645)	74,360 (1989)	27,819 (2704)	32,242 (2586)	52,242 (2094)	52,818 (2487)
*R*_work_/*R*_free_ (%)	19.1 (23.9)/23.8 (31.3)	20.1 (26.0)/23.6 (37.4)	19.5 (27.9)/21.6 (30.3)	19.2 (21.2)/24.5 (29.8)	19.2 (21.4)/23.1 (25.4)	20.0 (29.9)/22.4 (28.8)	20.3 (39.7)/27.5 (48.0)
No. of atoms							
Protein	3133	3142	3229	3182	3150	3288	13,207
MYR/EDO/MES/NA/CL/ZN/NO3/IOD	15/69/12/1/3/0/0	15/64/24/2/0/0/0	15/156/12/2/0/16/0	15/60/12/2/1/0/0	15/80/12/2/0/0/0	15/104/0/2/0/0/2/0	30/52/0/3/0/0/0/18
Water	81	92	273	162	189	214	34
*B*-factors (Å^2^)							
Protein	38.1	35.9	22.7	24.4	24.7	25.8	64.2
Ligand and ion	48.0	47.5	34.9	33.9	33.8	34.4	66.3
Water	36.6	35.8	27.8	25.4	25.8	28.8	45.1
RMSDs							
Bond lengths (Å)	0.010	0.009	0.009	0.010	0.009	0.007	0.009
Bond angles (°)	1.00	1.00	1.02	1.09	1.03	0.92	1.10
Ramachandran plot							
Favored (%)	97.6	98.7	99.2	98.1	99.2	98.9	94.1
Outliers (%)	0	0	0	0	0	0	0

PDB, Protein Data Bank.

a The highest resolution shell is shown in parentheses.

### Two basic conformations detected for lipopeptide ligands that were related to their differential antigenicity

Both crystal structures shown above indicated that the amide bond of Gly1 of the antigenic lipopeptides was exposed externally, enabling it to form a hydrogen bond with the side chain of E101 of the CDR3β loop. Therefore, we theorized that the differential antigenicity of C14nef4 and its five analogs may be related to the spatial position of Gly1 of each lipopeptide captured in the antigen-binding groove. To address this possibility, we determined a series of X-ray crystallographic structures of Mamu-B∗05104 complexed with either C14nef4 or each of the five lipopeptide analogs (Table 1). Protein structures in crystals may be affected by differences in crystallization conditions, such as pH, precipitants, and temperature (16). Therefore, we carefully completed crystallization procedures under identical conditions (Mes buffer, pH 6.5 with 20% polyethylene glycol [PEG] at 20 °C), except for C14-GGKI, for which a minor modification was added. The overall structure of Mamu-B∗05104 was virtually identical for all six complexes, with root-mean-square deviations calculated as ∼0.3 Å for all Cα atoms of the α1 and α2 domains between C14nef4 and its five analog-bound forms. Nevertheless, the spatial conformation of the lipopeptide ligands captured in the antigen-binding groove of Mamu-B∗05104 differed significantly between the highly and poorly antigenic groups.

**Figure 3:**
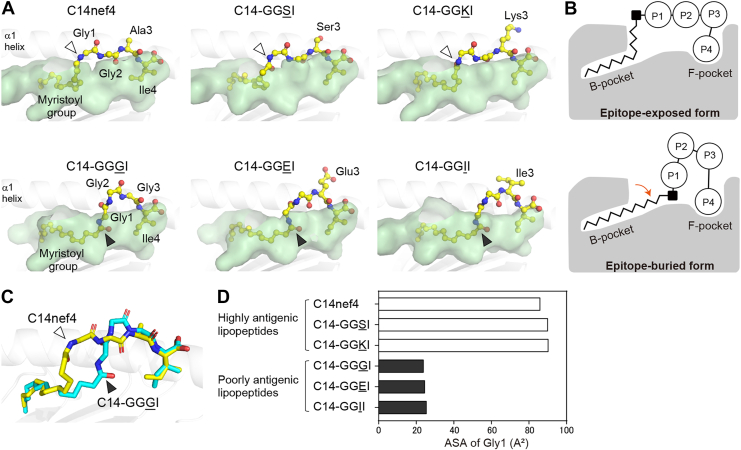
**Conformations of lipopeptides accommodated in the antigen-binding groove of Mamu-B∗05104.** The crystal structures of Mamu-B∗05104 complexed with the indicated lipopeptide ligands were determined, and ligand conformations were compared. *A*, lipopeptide ligands (*yellow sticks*) captured in the semitransparent antigen-binding groove (*green*) of Mamu-B∗05104 are shown. Note that the amide bond of Gly1 is exposed externally for highly antigenic ligands (*upper panels*, *open arrowheads*), whereas the epitopic structure is buried in the antigen-binding groove for poorly antigenic ligands (*lower panels*, *closed arrowheads*). *B*, two distinct conformations observed for highly (*upper panel*; referred to as the epitope-exposed form) and poorly (*lower panel*; referred to as the epitope-buried form) antigenic ligands are schematically illustrated. The T-cell epitopic amide bond of Gly1 is shown as *closed squares*. *C*, structures of the C14nef4 (*yellow*) and C14-GGGI (*cyan*) ligands obtained in (*A*) are superimposed with emphasis on the spatial shift of the T-cell epitopic amide bond of Gly1 (indicated with *open* and *closed arrowheads*, respectively). *D*, the ASA was calculated for the Gly1 residue of each lipopeptide ligand captured in Mamu-B∗05104 using the Shrake–Rupley algorithm with a solvent probe radius of 1.4 Å. ASA, accessible surface area.

The two anchor structures were similarly accommodated in the B- and F-pockets. The distal two-thirds of the fatty acids were captured in the B-pocket *via* van der Waals forces and hydrophobic interactions, whereas the C-terminal isoleucine residue (Ile4) was fixed in the F-pocket by forming a hydrogen bond network that was virtually identical among the six lipopeptide ligands (Figs. 3*A*, S2, and S3). However, the shape of the central portion of the lipopeptides differed significantly between the highly and poorly antigenic groups. The peptide stretch of the three N-terminal amino acid residues of the highly antigenic group (C14nef4, C14-GGSI, and C14-GGKI) was flat and extended horizontally above the antigen-binding groove, which was associated with vertical placement of the proximal part (C_1_–C_4_) of the linked fatty acid (Fig. 3*A*, *upper panels*, also see the *upper panel* in Fig. 3*B*). This resulted in placing the fatty acid–peptide junction, namely, the T-cell epitopic amide bond of Gly1, exposed externally (Fig. 3*A*, indicated with *open arrowheads*) at a distance reachable by the E101 residue of the CDR3β loop (2.6–3.0 Å) (Fig. S2). In contrast, the amide bond of Gly1 of the poorly antigenic group (C14-GGGI, C14-GGEI, and C14-GGII) shifted diagonally downward into the antigen-binding groove (Fig. 3*A*, *lower panels*, indicated by closed *arrowheads*), which was associated with horizontal placement of the proximal part (C_1_–C_4_ carbons) of the fatty acid and an archwise distortion of the central part of the peptide portion (Fig. 3, *A* and *B*, *lower panels*). The difference between these two basic conformations of the lipopeptide ligands was readily appreciable when the images of C14nef4 and C14-GGGI were superimposed (Fig. 3*C*). Importantly, the structural change detected for the poorly antigenic lipopeptides resulted in burial of the epitopic amide bond of Gly1 into the antigen-binding groove, making it unreachable by the CDR3β loop (5.8–5.9 Å) (Fig. S2). Consistent with this result, the accessible surface area (ASA) of Gly1 was significantly smaller in the poorly antigenic group than that in the highly antigenic group (Fig. 3*D*).

A side-by-side comparison of the six Mamu-B∗05104–lipopeptide complexes described above revealed two basic conformations of lipopeptide ligands: epitope-exposed and epitope-buried forms that were linked directly to their antigenicity (Fig. 3*B*). Furthermore, we also realized that the proximal portion (C_1_–C_4_ carbons) of the saturated fatty acid of lipopeptide ligands had substantial molecular flexibility (17, 18). This would be primarily because of the lack of double bonds and their limited interactions with surrounding structures. Thus, we reasoned that the epitopic amide bond of Gly1, which was directly linked to the proximal portion of the fatty acid, could dynamically change its spatial position, despite the pre-existing hydrogen bond network (Fig. S4). To address this issue directly, we sought to simulate the molecular dynamics of highly and poorly antigenic lipopeptide ligands.

### Molecular dynamics simulations of lipopeptide ligands

**Figure 4:**
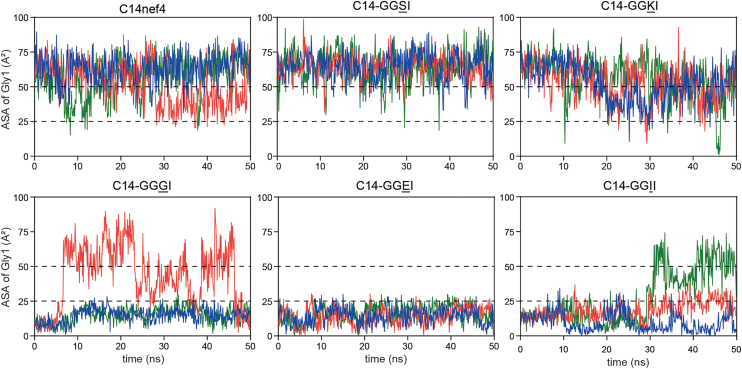
**Molecular dynamics simulations of lipopeptide-bound Mamu-B∗05104 complexes.** Molecular dynamics simulations were performed using the crystal structures of Mamu-B∗05104 complexed with either C14nef4 or each of its five analogs as initial models. Three independent 50 ns molecular dynamics simulations were conducted for each structure, and the ASA of Gly1 of the bound lipopeptides was monitored throughout the trajectories (5000 frames in total) at intervals of 10 frames. The results obtained from the three trajectories are shown in different colors (run 1, *blue*; run 2, *red*; and run 3, *green*). ASA values >50 Å^2^ and those <25 Å^2^ were provisionally considered to represent epitope-exposed and epitope-buried conformations, respectively. ASA, accessible surface area.

We performed molecular dynamics simulations for each crystal structure of the Mamu-B∗05104–lipopeptide complexes. The ASA value of the Gly1 residue of lipopeptide ligands was monitored because it is an excellent parameter related to exposure of the primary T-cell epitope and its antigenicity (Fig. 3*D*). Three independent 50 ns molecular dynamics simulations were run for each ligand, and molecular states with ASA values >50 Å^2^ and <25 Å^2^ were provisionally classified as epitope-exposed and epitope-buried conformations, respectively (Fig. 4). Among lipopeptide ligands of the highly antigenic group, C14-GGSI consistently sustained a molecular state with ASA values >50 Å^2^ during most of the measurement time in all three independent simulations (Fig. 4, *upper middle panel*). This contrasted sharply with the authentic C14nef4 antigen (*upper left panel*) and C14-GGKI (*upper right panel)*, which also formed an epitope-exposed conformation but were prone to occasionally shift into a transition state at ASA values between 50 Å^2^ and 25 Å^2^. These observations correlated with the results obtained from the T-cell–based and biolayer interferometry experiments, suggesting that the superb molecular stability exhibited by the C14-GGSI ligand accounts for its augmented antigenicity. As expected, lipopeptide ligands of the poorly antigenic group remained mostly in an epitope-buried state with ASA values below 25 Å^2^ (*lower panels*). However, an occasional shift into the transition state or even the epitope-exposed state was observed for C14-GGGI (*lower left*) and C14-GGII (*lower right*), presumably accounting for their limited antigenicity. Taken together, these observations indicate that lipopeptide ligands captured in antigen-binding grooves undergo dynamic conformational changes, and that their efficiency in sustaining the epitope-exposed form differs significantly and correlates directly with antigenic potential.

## Discussion

Conventional MHC class I–presented peptides typically comprise 8 to 11 amino acid residues, resulting in high degrees of sequence variations. These variations are precisely monitored in a highly specific manner using clonotypic TCRs selected from a repertoire of nearly infinite diversity (19). However, this fundamental concept of immunology may not be readily applicable to *N*-myristoylated short lipopeptides, which are also presented by MHC class I molecules. First, the peptide portion of MHC class I–presented lipopeptides is significantly shorter than that of conventional peptide antigens, as exemplified by the 4-mer lipopeptide C14nef4, which allows for only limited sequence variations. Second, the peptide sequence of lipopeptide antigens needs to conform to the basic motif (Gly-X-X-X-Ser/Thr) for protein *N*-myristoylation (20), which also limits their sequence variations. Last, TCRs recognize the amide bond of Gly1 without directly interacting with the peptide portion of C14nef4 (14). These lines of evidence suggest that, unlike conventional peptide-specific T cells, lipopeptide-specific T cells may not precisely monitor sequence variations in the peptide portion of lipopeptide antigens. A small amount of *N*-myristoylated lipopeptides may be generated in healthy cells (21, 22); however, N-terminal fragments of *N*-myristoylated viral proteins accumulate abundantly in virus-infected cells during a short period after infection (23). Therefore, it is reasonable to consider that lipopeptide-specific T cells monitor the quantity of *N*-myristoylated lipopeptides within the target cells rather than monitoring the quality, namely, their sequence variations precisely.

Nevertheless, the C14nef4 lipopeptide ligand and its analogs with an amino acid substitution for Ala3 can be separated into highly and poorly antigenic groups. The present study detected two basic conformations: epitope-exposed and epitope-buried forms for the highly and poorly antigenic lipopeptides, respectively. Importantly, these conformations correlated with their differential antigenicity. In both forms, the amide bond of Gly1 was fixed by a unique hydrogen bond network (Fig. S4). However, the proximal portion of the hydrocarbon chain protruded outside the B-pocket and exhibited significant plasticity, which appeared to affect the spatial position of the directly linked amide bond of Gly1. It is noteworthy that some lipopeptide ligands can potentially switch their conformations depending on the crystallization conditions and access of TCRs (24). Pertinent examples include C14nef4, for which both conformations have been detected using X-ray crystallography (9, and in this study). More importantly, the hydrogen bond network involving the amide bond of Gly1 can be dynamically reorganized upon TCR docking. This induced-fit mechanism supports a shift from epitope-buried to epitope-exposed forms (14).

No apparent chemical or structural properties of the amino acid residue at position 3 are shared by either the highly or poorly antigenic groups. Moreover, the potential effect of amino acid selection at this position on lipopeptide conformation and antigenicity remains unclear. We theorized that this effect is multifaceted, depending on the side-chain size, polarity, and dihedral angle flexibility (25). For example, the selection of glycine at this position may confer a high degree of structural variation because the simplest hydrogen side chain allows for significant rotational freedom of the peptide chain (26). Rotation of the carbonyl oxygen of the Gly2 residue of C14-GGGI appeared to support the curved deformation of the peptide chain (Figs. 3*C* and S4). In the case of C14-GGEI and C14-GGII, molecular interactions between Glu3/Ile3 and surrounding residues of Mamu-B∗05104 may primarily determine the shape of the peptide chain.

Non-MHC–encoded molecules of the CD1 family possess hydrophobic pockets that accommodate a variety of lipids and glycolipids and activate lipid-specific T cells (27). Several lines of evidence indicate that CD1 molecules can also mediate lipopeptide–antigen presentation. For example, human CD1a and CD1c isoforms have been reported to bind a mycobacterial C20 lipopeptide, dideoxymycobactin (28) and a synthetic C16-palmitoylated 12-mer lipopeptide (29), respectively, and present them to specific T cells. In addition, mouse CD1d can capture a C16-modified 22-mer lipopeptide that adopts α-helical conformation (30). The crystal structures of lipopeptide-bound human CD1a and mouse CD1d complexes show that acyl chains are buried deeply within the hydrophobic pockets of CD1 molecules, leaving only the peptide portion exposed externally. Fine molecular mechanisms underlying TCR recognition of these CD1-bound lipopeptides are yet to be elucidated. Nevertheless, these structures suggest that the peptide moiety of the CD1-bound lipopeptides may play a pivotal role in TCR recognition, as is the case for conventional peptide antigens presented by MHC class I molecules.

Activation of T-cell populations with potent antigens results in robust expansion of effector T cells and memory-type T cells (31, 32), which provides a basis for efficient and sustained host protection. Otherwise, insufficient TCR signaling induced by imperfect antigens may lead to undesirable immune conditions, referred to as T-cell anergy and exhaustion (33). Furthermore, autoimmunity can be induced by molecular mimicry mechanisms, in which T cells primed by foreign antigens crossreact with structurally related self-antigens (34). Although extensive studies have delineated these principles in the context of peptide–antigen presentation, the immunological mechanisms of lipopeptide-specific T-cell responses are only beginning to be understood. In addition, how lipopeptides with limited molecular variations can contribute effectively to host defense remains to be elucidated. We recently determined the crystal structures of the HLA-A∗24:02 and HLA-C∗14:02 complexes that bound C14-Gly1-Ala2-Asn3-Phe4 and C14-Gly1-Ala2-Ala3-Leu4 lipopeptides, respectively (10). These 4-mer lipopeptides exhibited “epitope-exposed” conformations in the antigen-binding groove, although T-cell responses to these lipopeptides have yet to be monitored. Our findings obtained from the present study support the possibility that lipopeptide-specific T-cell responses may indeed exist in humans.

## Experimental procedures

### Recombinant proteins

Recombinant proteins were prepared as previously described (8). Briefly, the ectodomain of the Mamu-B∗05104 heavy chain, rhesus β2m, and the ectodomains of SN45 TCRα and β chains were expressed in *E. coli* as inclusion bodies, and the purified inclusion bodies were dissolved in 6 M guanidine–HCl. The Mamu-B∗05104 heavy chain and β2m proteins were then refolded in the presence of each synthetic lipopeptide (provided by GenScript) in 500 ml of refolding buffer (100 mM Tris–HCl, pH 8.3, 500 mM l-arginine, 2 mM EDTA, 0.5 mM oxidized GSH, and 5 mM reduced GSH) for 48 h at 10 °C. The SN45 TCRα and β proteins were mixed in 1.5 l of refolding buffer (100 mM Tris–HCl, pH 8.1, 400 mM l-arginine, 2 mM EDTA, 5 M urea, 3.7 mM cystamine, and 6.6 mM cysteamine) and incubated for 24 h at 10 °C. After dialysis against 10 mM Tris, pH 8.0, the refolded proteins were concentrated and purified sequentially using Superdex 200 Increase size-exclusion chromatography (GE Healthcare), followed by monoQ anion exchange chromatography (GE Healthcare). The TCRβ chain fused with the C-terminal Avi-tag sequence (LHHILDAQKMVWNHR) was prepared for use in biolayer interferometry experiments. The purified TCR protein was biotinylated using the BirA enzyme (Sigma–Aldrich) and further subjected to size-exclusion chromatography to remove free biotin.

### Biolayer interferometry binding experiments

Biomolecular interaction analyses were performed using the Octet RED96 system equipped with a streptavidin-conjugated biosensor (Sartorius), according to the manufacturer’s instructions. The biotinylated SN45 TCR protein was immobilized on the sensor tip surface, with a signal magnitude of approximately 2.5 nm. The sensors were treated with 5 μg/ml biocytin for quenching, incubated with 1:2 dilution series of lipopeptide-bound Mamu-B∗05104 proteins starting at 40 μM for 60 s (for association), and incubated in analyte-free assay buffer (10 mM Tris–HCl, pH 7.4, and 100 mM NaCl) for 60 s (for dissociation). A shift in the interference pattern was monitored in real time during this period. After the reference subtraction, the equilibrium dissociation constant (*K*_*D*_) was determined using the global curve-fitting method.

### X-ray crystallographic analysis

One microliter of the highly purified lipopeptide-bound Mamu-B∗05104 proteins (10 mg/ml) was mixed with a mother liquid containing 100 mM Mes buffer, pH 6.5 and 20% PEG Smear High (a mixture of PEG 6000, 8000, and 10,000, supplied by Molecular Dimensions Ltd) for Mamu-B∗05104 complexed with either C14nef4, C14-GGSI, C14-GGGI, C14-GGEI, or C14-GGII lipopeptides. For the Mamu-B∗05104–C14-GGKI complex, the mother liquid was supplemented with 0.2 M NH_4_NO_3_ salt. All crystals were formed at 20 °C using the sitting-drop vapor diffusion method. For the Mamu-B∗05104–C14-GGSI–SN45 TCR complex, the C14-GGSI-bound Mamu-B∗05104 and SN45 TCR proteins were mixed at a molar ratio of 1:1 and incubated at room temperature for 1 h to induce formation of the ternary complex. Then, 1.5 μl of the solution (10 mg/ml) was mixed with 1.5 μl of a mother liquid containing 0.2 M sodium iodide, 0.1 M Bis–Tris–propane at pH 6.25 and 17.5% PEG3350. Crystallization was then allowed to proceed at 20 °C using the hanging-drop vapor diffusion method. Diffraction data were collected at 100 K (in a cold nitrogen gas stream) on an EIGER X 4M detector (DECTRIS) using synchrotron radiation with a wavelength of 1.0 Å at the BL26B1 station (SPring-8; Hyogo). The resulting datasets were processed, merged, and scaled using XDS (https://xds.mr.mpg.de/) (35). Structures were solved by Phase-MR implemented in PHENIX 2.0 (https://www.phenix-online.org/) (36) with previous structures (Protein Data Bank ID: 6IWG for the binary complexes and Protein Data Bank ID: 7BYD for the ternary complex) as search models. The model was refined using the phenix.refine. The structures were rebuilt using COOT 0.9.8 (https://www2.mrc-lmb.cam.ac.uk/personal/pemsley/coot/) (37) and further modified based on σ-weighted (2|Fo|—|Fc|) and (|Fo|—|Fc|) electron density maps. Crystallographic images were obtained using the PyMOL software (DeLano Scientific).

### Molecular dynamics simulations

Molecular dynamics simulations were performed as previously (38). The crystal structures of each Mamu-B∗05104–lipopeptide complex were used as the initial structural data. Flexible side chains were modeled using the structure preparation module in molecular operating environment (MOE). The dominant protonation states at pH 7.0 were assigned to titratable residues. For each complex, three independent molecular dynamics simulations of 50 ns were performed with different atomic velocities using the AMBER 24 package (39) and amber10:EHT force field. Each system was solvated in a TIP3P (40) water box extending 10 Å beyond the protein surface and relaxed using the default relaxation protocol implemented in MOE. Simulations were performed in the NPT ensemble at 300 K and 100 kPa, with the temperature and pressure controlled by a Langevin thermostat and Monte Carlo barostat, respectively. van der Waals and short-range electrostatic interactions were assigned using a cutoff of 8 Å, and long-range electrostatic interactions were treated using the smooth particle mesh Ewald method (41). ASA values were calculated using the Shrake–Rupley algorithm implemented in MOE with a probe radius of 1.4 Å. The ASA values for the Gly1 residues of each lipopeptide were obtained from trajectory snapshots captured every 10 frames throughout the 5000-frame trajectory.

## Data availability

The datasets used and analyzed in the study are available upon request.

## Supporting information

This article contains supporting information.

## Conflict of interest

The authors declare that they have no conflicts of interest with the contents of this article.
