# Supplementary material for: Lipopeptide ligands captured by MHC class I molecules undergo dynamic conformational changes that affect their antigenic strength
Source: J Biol Chem. 2025 Dec 12;302(2):111049. doi: 10.1016/j.jbc.2025.111049 (PMC12804144; doi:10.1016/j.jbc.2025.111049)
Supplement: Supplemental Figures [file mmc1.pdf]

Lipopeptide ligands captured by MHC class I molecules undergo dynamic conformational changes that affect their antigenic strength

Daisuke Morita[1*], Toshiki Fujii[2], Shinsuke Inuki[2,3,4], Hiromu Suzuki[1,5], Bunzo Mikami[6,7], and Masahiko Sugita[1*]

[1]Laboratory of Cell Regulation, Institute for Life and Medical Sciences, Kyoto University, Kyoto, Japan.

[2]Laboratory of Bioorganic Medicinal Chemistry, Graduate School of Pharmaceutical Sciences, Kyoto University, Kyoto, Japan.

[3]Laboratory of Bioorganic Medicinal Chemistry, Graduate School of Biomedical Sciences, Tokushima University, Tokushima, Japan.

[4]Laboratory of Medicinal Chemistry, Institute of Photonics and Human Health Frontier, Tokushima University, Tokushima, Japan.

[5]Laboratory of Cell Regulation and Molecular Network, Graduate School of Biostudies, Kyoto University, Kyoto, Japan.

[6]Laboratory of Metabolic Science of Forest Plants & Microorganisms, Research Institute for Sustainable Humanosphere, Kyoto University, Kyoto, Japan.

[7]Structural Energy Bioscience, Institute of Advanced Energy, Kyoto University, Kyoto, Japan.

Supporting information: Figures S1-S4

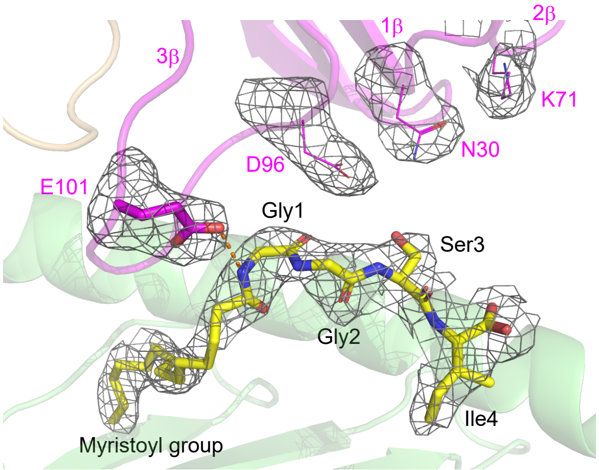

SN45 TCR			C14-GGSI		Distance	Hydrogen bonds
CDR3β	E101	OE2	N	Gly1	2.8	+
CDR1β	N30	ND2	OG	Ser3	3.8	
CDR3β	D96	OD1			4.3	
		OD2			4.1	
CDR2β	K71	NZ			9.4	

Figure S1. Electron density map at the TCR/ligand interface of the SN45 TCR:C14-GGSI:SN45 TCR complex. The Fo–Fc polder omit map generated by Phenix (contoured at 3.0σ) for the C14-GGSI lipopeptide (yellow) and the side chains of nearby amino acid residues of the CDR1β, CDR2β and CDR3β loops (magenta) are shown as gray mesh with a hydrogen bond indicated with a dotted line (left panel). Distances between the lipopeptide ligand and nearby amino acid residues in the TCR loops are listed in the right panel. Hydrogen-bond contacts were defined using a distance cut-off of 3.4 Å.

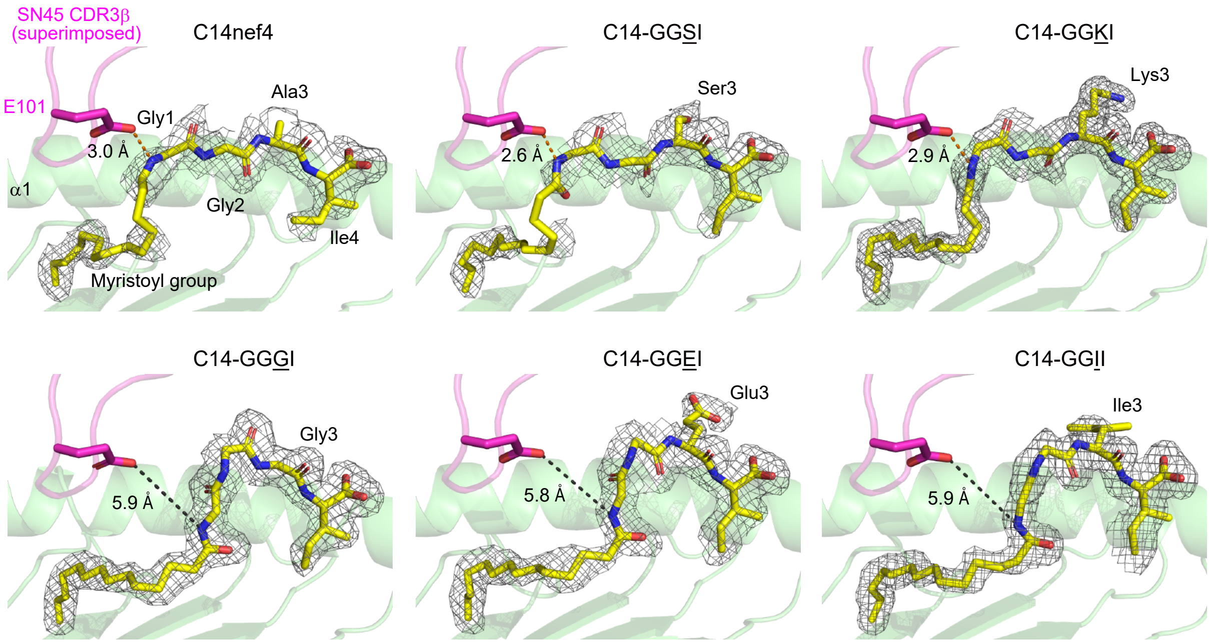

Figure S2. Electron density maps of lipopeptides ligands in the Mamu-B*05104:lipopeptide complexes. The Fo–Fc polder omit maps generated by Phenix (contoured at 3.0σ) for the indicated lipopeptide ligands are shown (gray mesh). The bound lipopeptides (yellow sticks) and α1 helical structure (green ribbons) of Mamu-B*05104 are also displayed. Based on the crystal structure of the Mamu-B*05104:C14nef4:SN45TCR complex, the CDR3β loop (magenta) of SN45 TCR is provisionally superimposed to estimate the distance (dotted lines) between the side chain of E101 of the CDR3β loop and the amide bond of Gly1 of each lipopeptide.

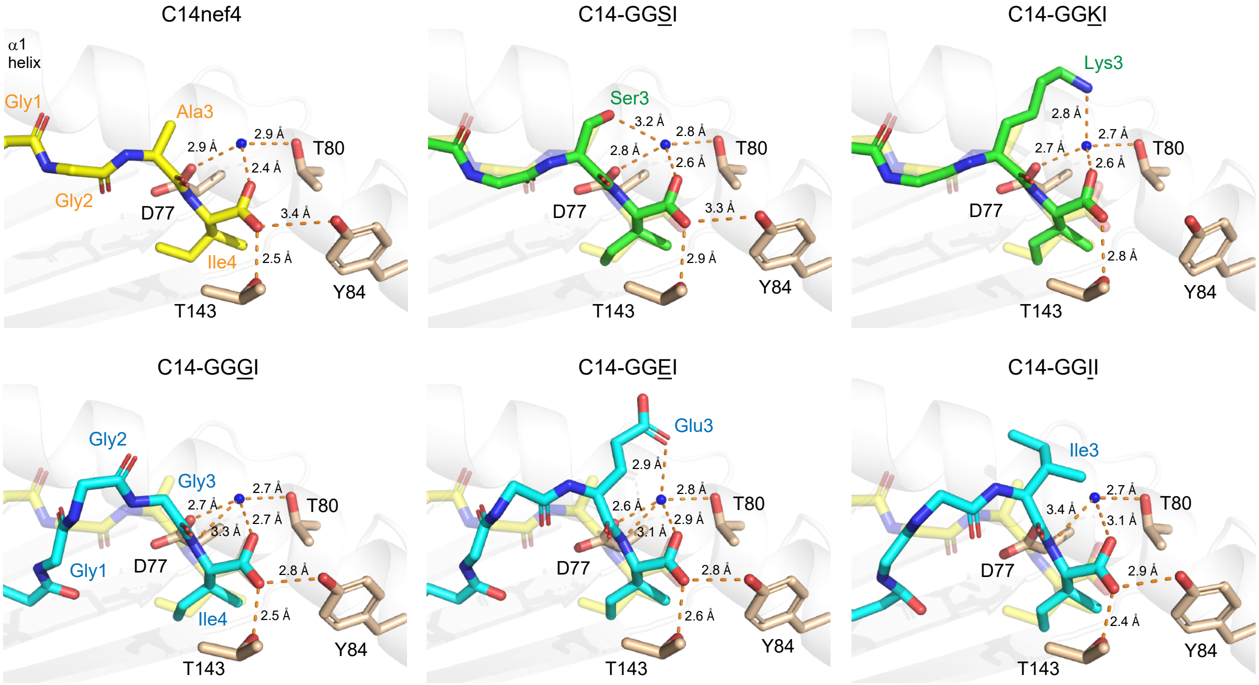

Figure S3. Hydrogen-bond networks established for the C-termini of lipopeptide ligands in lipopepde-bound Mamu-B*05104 complexes. Side views of the C-termini of the indicated lipopeptides are shown. Amino acid residues that form hydrogen bonds (orange dashed lines) with the C-terminal Ile residue of each lipopeptide are displayed as light brown sticks. Water molecules are shown as blue spheres. In the panels for the highly (green) and poorly (cyan) antigenic analogs, the configuration of C14nef4 (yellow semitransparent sticks) is superimposed.

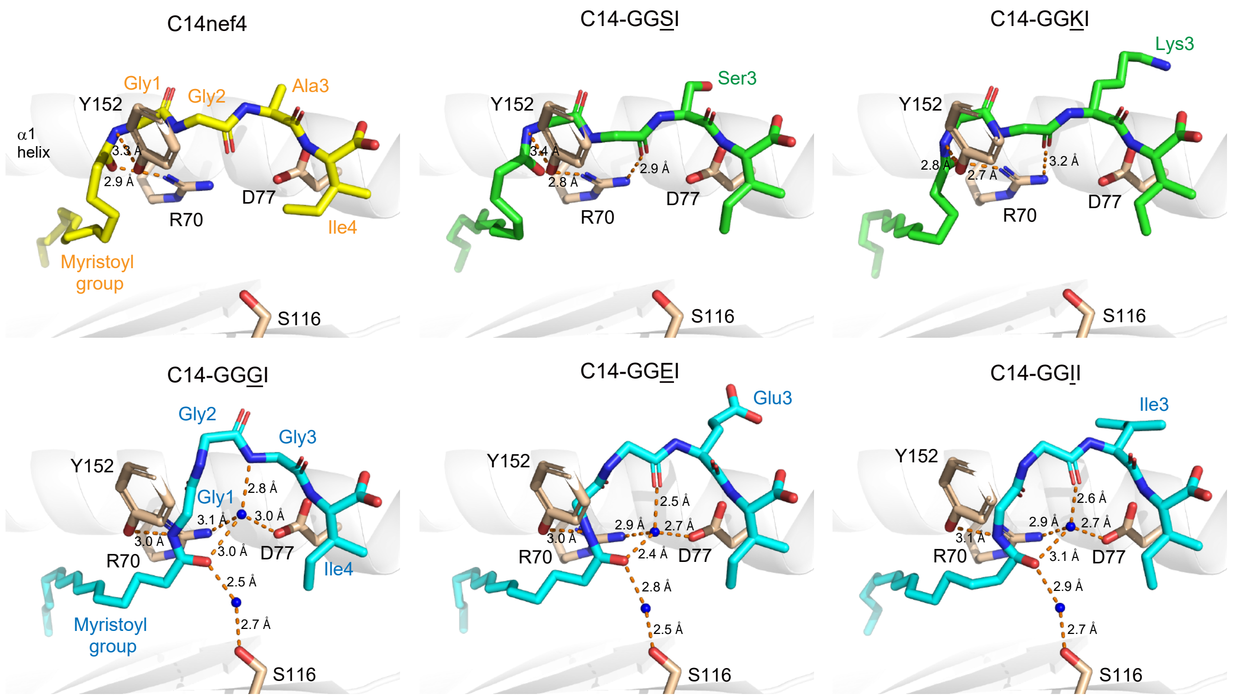

Figure S4. Hydrogen-bond networks established for the amide bond of Gly1 of lipopeptide ligands in lipopeptide-bound Mamu-B*05104 complexes. Side views of the indicated lipopeptides are shown. Amino acid residues that form hydrogen bonds (orange dashed lines) with the amide bond of Gly1 in each lipopeptide are shown as light brown sticks. Water molecules are shown as blue spheres.
